# Clinical Outcomes of Cryo Nerve Ablation Technique for Pain Management: An Exploratory Study in Patients Undergoing Left Thoracotomy Coronary Artery Bypass Grafting

**DOI:** 10.31083/j.rcm2406182

**Published:** 2023-06-25

**Authors:** Aleksander Dokollari, Serge Sicouri, Ozgun Erten, Basel Ramlawi, Matteo Cameli, Giulia Elena Mandoli, Beatrice Bacchi, Francesco Cabrucci, Francis P. Sutter, Stephanie Kjelstrom, Georgia Montone, Noah Sicouri, Leila Hosseinian, Massimo Bonacchi, Gianluca Torregrossa

**Affiliations:** ^1^Department of Cardiac Surgery Research, Lankenau Institute for Medical Research, Main Line Health, Wynnewood, PA 3450, USA; ^2^Department of Cardiac Surgery, Lankenau Heart Institute, Lankenau Medical Center, Main Line Health, Wynnewood, PA 3450, USA; ^3^Department of Cardiology, Le Scotte Hospital, University of Siena, 53100 Siena, Italy; ^4^F.U. of Cardiac Surgery, Department of Experimental and Clinical Medicine - University of Florence, 50134 Firenze, Italy; ^5^Department of Cardiac Surgery, Population Health, Lankenau Institute for Medical Research, Wynnewood, PA 3450, USA

**Keywords:** cryoICE™, Cryo Nerve Block, pain, numbness, sensitivity, opioids

## Abstract

**Background:**

To investigate the impact of Cryo Nerve Block with cryoICE™ device utilization, on post-operative pain in patients undergoing isolated coronary artery bypass grafting (CABG) through left thoracotomy.

**Methods:**

All consecutive patients undergoing isolated CABG through left thoracotomy between July 2021 and July 2022 from a single surgeon were included in the study. Patients using the cryoICE™ device for nerve block were compared for baseline demographics and pre-operative characteristics with those that did not use the cryoICE™ device. A propensity-adjusted analysis was used to compare the two groups. The primary outcome was degree of incisional pain and numbness.

**Results:**

A total of 103 patients underwent isolated CABG through left thoracotomy. After matching, the cryoICE™ device was used for nerve block in 60 patients while the control group included 43 patients. Mean follow-up was 5.7 months. The cryoICE™ device group had a mean value of incisional pain at hospital discharge was 1.5 (scale 0–10) while at follow-up was 0.69 (scale 0–10). Mean values of skin numbness at hospital discharge were 1 (scale 0–10) and 0.57 (scale 0–10) at follow-up. After univariate analysis comparison of cryoICE™ device group (60 patients) versus non-cryoICE™ device group (43 patients), the total in-hospital morphine use was 49% lower in the cryoICE™ versus the non-cryoICE™ cohort (73.8 + 79.37 mg vs 144.1 + 118.99 mg).

**Conclusions:**

Good clinical outcomes were observed in patients undergoing isolated left thoracotomy CABG with cryoICE™ utilization, including a very low incidence of post-operative pain, numbness, and hypersensitivity for all comers.

## 1. Introduction

Post-operative pain management after cardiac surgery produced a florid and constructive debate due to the increasing rate/costs of opioids overdose [1, 2]. Minithoracotomy consists in opening spreading muscles and ribs and opening the pleural space, therefore causing a major trauma that leads to a higher intensity of post-operative pain when compared to ministernotomy surgical procedures [3]. High levels of post-operative pain are associated with cardiovascular and respiratory complications, and proper pain management is crucial for enabling fast recovery [3, 4].

The cryoICE™ study for pain management in post thoracic procedures via intercostal cryoanalgesia (FROST) clinical trial reported improved outcomes of pain management utilizing the cryoICE™ device for patients undergoing minimally invasive valve surgery [5]. However, concerns remain regarding the persistence of pain, skin numbness and hypersensitivity after the surgical procedure. Despite the benefits shown with Cryo Nerve Block, its effects on post-operative physical function and psychological status of patients, considered to be equally important, remain unknown. In addition, the process of harvesting of the left internal thoracic artery (LITA) can be associated with severe post-operative pain due to either nerve inflammation, or skin numbness [6].

The main goal of this study is to investigate the efficacy of Cryo Nerve Block using the cryoICE™ (Atricure, Mason, OH, USA) device, on post-operative pain in patients undergoing isolated coronary artery bypass grafting (CABG) through left thoracotomy, utilizing visual-pain questionnaires. In addition, we investigated skin hypersensitivity and persistence of skin numbness following surgery.

## 2. Methods

### 2.1 Study Population

All consecutive patients undergoing isolated CABG through left thoracotomy between July 2021 and July 2022 from a single surgeon at Lankenau Medical Center, PA, USA, were included in the study. Patients using the cryoICE™ device for nerve block were compared for baseline demographics and pre-operative characteristics with those that did not use the cryoICE™ device. During the first 5-months of the surgeon (GT) clinical practice, he did not use the cryoICE™. After that period, he introduced the cryoICE™ device in his clinical practice for all patients undergoing isolated left thoracotomy CABG. Patients were identified via operation codes in a digital operation registry, as well as from a centralized cardiac surgery database for all CABG operations. Clinical data were collected retrospectively from medical records. The underlying in-hospital outcomes were recorded from the charts and death certificate made out by the responsible physician. Follow-up of patients was performed at our outpatient’s clinic, from the hospital registry and by telephone call (IRB E-22-5266). Inclusion criteria were consecutive patients that underwent isolated CABG through left thoracotomy. Exclusion criteria were patients with a concomitant cardiac procedure and that did not use cryoICE™, cardiac surgery via full sternotomy, pregnancy, current use of opioids, myocardial infarction (MI) within 30 days of informed consent, history of substance abuse, chronic pain syndrome, and psychiatric, physical, or mental conditions that would interfere with pain assessment. A psychiatric evaluation was performed by a psychiatrist who made an overall assessment of the patients and provided insights on whether a patient was mentally suitable for this type of procedure.

At follow-up, we investigated in-hospital and follow post-operative pain in the post-operative period, with particular attention on skin hypersensitivity and persistence of skin numbness, utilizing visual-pain questionnaires.

The study protocol was approved by the Main Line Health Hospitals Institutional Review Board (IRB 45CFR164.512).

### 2.2 Primary and Secondary Goals, Definitions and Data Collection

Primary outcome was grade of incisional pain and numbness. Secondary outcomes were all cause mortality, major adverse cardiovascular and cerebrovascular events (MACCE), nonfatal stroke, nonfatal MI, and repeat intervention. Pre-operative definitions and post-operative outcomes definitions followed the Society of Thoracic Surgeons definitions (**Supplementary Document 1**). Anesthesia management and covariates and exposures are included in **Supplementary Document 2**. A description of the cryoICE™, surgical indications, and complications according to the company’s suggestions are described in **Supplementary Document 3**. Patients’ questionnaire was added in **Supplemental Document 4**. Only 1 surgeon (GT) performed all the operations. A detailed description of the surgical procedure is added in **Supplementary Document 2**.

### 2.3 Anesthetic Management

Anaesthetic management was similar in all patients, and included standard monitoring techniques (electrocardiography, central venous/pulmonary artery and arterial pressure monitoring, urinary output, and nasopharyngeal and urinary bladder temperature monitoring).

Standardized intravenous (IV) and oral opioids were used for pain management during the postoperative period as part of institutional program. Intravenous fentanyl was used in patients in the operating room. Ketorolac was also used in the operating room and in the management of post-operative pain. Oral oxycodone was used in the post-operative period for pain management. We used local injections of lidocaine at the surgical incision site in the operating room.

### 2.4 CryoICE™ Surgical Procedure

Cryo nerve block consists in temporarily blocking intercostal nerve conduction under each rib. After the procedure, the patient may experience a sensation of numbness in the incision site which translates in decreased pain. The probe is inserted in the thoracotomy incision, the intercostal nerve that innervates the surgical incision site is localized, and the cryosphere is placed adjacent to the intercostal bundle (nerve-artery-vein) close to the innermost intercostal muscle in a location proximal to the latero-cutaneous nerve branch, at a distance of at least 2 cm from the ganglia and 4 cm from the base of the spine. We maintain gentle probe pressure on the tissue throughout cryoablation period. Cryoablation is performed for 120 seconds at –65 °C at 2 levels above the incision, at the level of the incision and 2 levels below the lowest incision.

None of the authors have received funding related to this study and none of the authors are affiliated to the company which produces the Cryonerve device.

### 2.5 Post-Operative Pain Management

Rescue analgesia was defined as hydromorphone or oxycodone administration if pain intensity was higher than 5 (based on a scale from 1 to 10 where 1 is the lowest intensity of pain and 10 is the most severe pain), or if pain persisted after administration of acetaminophen and gabapentin. In the post-operative, 1 gr IV acetaminophen is administered for pain management every 4 hours with a maximum dose of 3 gr. If pain persists, oral gabapentin 300 mg is administered up to three times per day, after patient mental status assessment looking for signs of delirium. In case the pain persisted, hydromorphone 0.2 mg IV every 2 to 3 hours, as needed was administered up to a maximum of 3 mg per day. Next step, in case of continuous pain, was administration of oral oxycodone 5 mg as needed. In case creatinine clearance was <30 mL/min 50% of the usual oxycodone dose was used. In both study arms, standardized IV and oral opioids were used for pain management during the post-operative period as part of an institutional protocol (**Supplementary Document 2**). In our analysis, we collected and presented the cumulative, intra-operative and post-operative amount of opioid use. A second cohort undergoing the same surgical procedure from the same surgeon without cryoICE™ was used as a control group for in-hospital opioids use. The conversion rate of opioids to morphine milligram equivalent was done following the American Pain Society guidelines [7]. For simplicity we added the conversion table in **Supplementary Document 2**.

### 2.6 Statistical Analyses

We evaluated each demographic and pre-operative variable as a risk factor for clinical outcomes of all-cause mortality, MACCE, stroke, MI, reoperation, and angina. Groups were compared by two-sample *t*-tests or Wilcoxon Rank Sum Test for continuous variables and chi-square test of independence for categorical variables. A propensity-adjusted matching was used via a multiple logistic regression with cryoICE™ as the dependent variable and all demographics and pre-operative variables added to the model. A 1:1 greedy nearest neighbor with no replacement match and caliper width of 0.2 produced two groups, with cryoICE™ (N = 60 patients) and non-cryoICE™ (N = 43 patients). Success of matching was assessed by computing the percent bias (similar to standardized mean difference) of each covariate with a cut-off of 10% to denote acceptable balance. Matched samples were compared with McNemar’s test and marginal homogeneity tests for categorical variables and matched paired *t*-tests and signed rank tests for continuous variables. This analysis allowed inclusion of patients who died within the first 30-days of surgery and was performed to account for possible immortal time-bias. All analyses were performed in Stata 17.0 (Statacorp, LLC. College Station, TX, USA).

### 2.7 Follow-Up Outcomes

Study patients were followed in the post-operative period and at hospital discharge. All patients were contacted after surgery by telephone to screen for hyperalgesia using a visual-pain questionnaire (**Supplementary Document 4**). In case of a positive response, the patient was asked to make an office visit for a more thorough allodynia assessment. The cotton tip applicator test was used, a test previously validated as a reproducible screening method to identify cutaneous allodynia [5].

## 3. Results

### 3.1 Pre-Operative Characteristics

There was a total of 103 patients undergoing isolated left thoracotomy CABG, of whom 60 patients received cryoICE™ and 43 patients did not. Mean follow-up period was 5.7 months.

After matching 14 pre-operative variables, diabetes, and chronic obstructive pulmonary disease (COPD) were significantly higher in the cryoICE™ cohort. The cryoICE™ cohort (Table 1) had a mean age of 69.6 ± 9.5 years and a mean STS score (%) of 1.4 ± 1.1 while, the non- cryoICE™ had a mean population age of 67.5 ± 10.1 years and a mean STS score was 1.2 ± 1.1%.

**Table 1.** **Propensity-adjusted pre-operative characteristics**.

Pre-operative characteristics		cryoICE™ patients	Non-cryoICE™	*p*-value	All patients
		N = 60	N = 43		N = 103
Gender male, n (%)		47 (78.3%)	38 (90.5%)	0.105	85 (83.3%)
Age years, mean ± SD		69.6 ± 8.9	67.5 ± 10.1	0.275	68.7 ± 9.4
STS risk score %, mean ± SD		1.9 ± 3.8	1.2 ± 1.1	0.261	1.6 ± 3.03
Creatinine level, mean ± SD		1.03 ± 0.2	1.12 ± 0.3	0.079	1.07 ± 0.3
Smoking status, n (%)				0.668	
	Former smoker	22 (37.3%)	17 (40.5%)		39 (38.6%)
	Active smoker	7 (11.9%)	4 (9.5%)		11 (10.9%)
Diabetes, n (%)		14 (23.7%)	24 (57.1%)	0.001	38 (37.6%)
Chronic obstructive pulmonary disease, n (%)		14 (23.3%)	3 (7.1%)	0.034	17 (16.7%)
Hypertension, n (%)		55 (91.7%)	40 (95.2%)	0.483	95 (93.1%)
Cerebrovascular events, n (%)		17 (28.3%)	10 (23.8%)	0.610	27 (26.5%)
Peripheral vascular disease, n (%)		9 (15%)	8 (19.05%)	0.589	17 (16.7%)
Prior cardiac surgery, n (%)		2 (3.3%)	0	0.232	2 (2.0%)
Prior PCI, n (%)		20 (33.3%)	16 (38.1%)	0.620	36 (35.3%)
Atrial fibrillation, n (%)		8 (13.3%)	6 (14.6%)	0.853	14 (13.9%)
Myocardial infarction, n (%)		32 (53.3%)	17 (40.5%)	0.201	49 (48.0%)
EF %, mean ± SD		58.9 ± 11.2	57.5 ± 10.9	0.536	58.3 ± 11.0
Number of diseased vessels				0.063	
	One, n (%)	10 (16.7%)	4 (9.5%)		14 (13.7%)
	Two, n (%)	27 (45%)	12 (28.6%)		39 (38.2%)
	Three, n (%)	23 (38.3%)	26 (61.9%)		49 (48.0%)

PCI, percutaneous coronary intervention; EF, ejection fraction.

### 3.2 Intra-Operative Outcomes

Intraoperatively (Table 2), there were no differences among the two groups. Both groups had off-pump CABG, and LITA anastomosis to the left anterior descending (LAD) artery was used in 100% of cases. The radial artery graft was used in 1 (1.7%) patient in the cryoICE™ cohort and 5 (11.9%) patients in the non-cryoICE™ one. In addition, there were 3 (5%) saphenous venous grafts anastomoses in the cryoICE™ cohort and 1 (2.4%) in the non-cryoICE™ cohort, while 51 (85%) patients and 36 (83.7%) were extubated in the OR in the cryoICE™ and cryoICE™ cohorts, respectively. There was no intra-operative death but there was a non-lethal stroke from which the patient fully recovered.

**Table 2.** **Intra-operative outcomes**.

Intra-operative outcomes	cryoICE™ patients	Non-cryoICE™ Patients	*p*-value	All patients
	N = 60	N = 43		N = 103
OR time (hours), mean ± SD	5.6 ± 0.93	5.5 ± 0.94	0.931	5.5 ± 0.93
RBCs transfusion units, n (%)	2 (3.3%)	2 (4.6%)	0.715	4 (3.9%)
Cryoprecipitate transfusion unit, n (%)	1 (1.7%)	0	0.400	1 (1.0%)
LITA use, n (%)	60 (100%)	43 (100%)	1.000	103 (100%)
Total distal anastomosis with arterial graft conduit, n (%)	61 (100%)	48 (100%)	1.000	109 (100%)
Total distal anastomosis with venous graft conduit, n (%)	3 (5%)	1 (2.3%)	0.451	4 (4.1%)
Total distal anastomosis with radial artery graft conduit, n (%)	1 (1.7%)	5 (11.6%)	0.081	6 (6.2%)
Extubation in OR, n (%)	51 (85%)	36 (83.7%)	0.745	87 (84.4%)
Intra-operative deaths, n (%)	0	0	1.000	0

RBCs, red blood cells; OR, operating room; LITA, left internal thoracic artery.

### 3.3 Post-Operative Outcomes

Postoperatively, there were no differences among groups. Ventilation time was 1.05 ± 3.42 vs 0.45 ± 1.28 hours, in the cryoICE™ versus cryoICE™ cohorts, respectively, while no patient had prolonged ventilation time (≥24 hours). Total hospital length of stay (LOS) was 4.1 ± 2.6 vs 4.1 ± 1.9 days in the cryoICE™ versus non-cryoICE™ cohorts, respectively (Table 3). One patient had a reoperation for bleeding in the cryoICE™ group, and there was no in-hospital death.

**Table 3.** **Post-operative outcomes**.

Post-operative Outcomes	cryoICE™ Patients	Non-cryoICE™ Patients	*p*-value	All patients
	N = 60	N = 43		N = 103
Ventilation time (hours), mean ± SD	1.05 ± 3.42	0.45 ± 1.28	0.284	0.8 ± 2.73
Prolonged ventilation hours ≥24 hours, mean ± SD	0	0	1.000	0
Stroke, n (%)	1 (1.7%)	0	0.400	1 (0.9%)
Infection, n (%)	0	0	1.000	0
Reoperation for bleeding, n (%)	1 (1.7%)	0	0.392	1 (1.0%)
Creatinine level, mean ± SD	1.23 ± 0.47	1.34 ± 0.52	0.271	1.28 ± 0.49
New atrial fibrillation n (%)	17 (29.3%)	16 (38.1%)	0.356	33 (33.0%)
RBCs transfusion units, n (%)	5 (8.6%)	4 (9.3%)	0.944	9 (8.7%)
Cryoprecipitate transfusion units, n (%)	2 (3.4%)	0	0.224	2 (2.0%)
FFP transfusion units, n (%)	2 (3.4%)	0	0.224	2 (2.0%)
In-hospital deaths, n (%)	0	0	1.000	0
Total Length of stay (days) mean ± SD	4.1 ± 2.6	4.1 ± 1.9	0.986	4.1 ± 2.3

RBCs, red blood cells; FFP, fresh frozen plasma.

### 3.4 Follow-Up Outcomes

At follow-up, there were no significant differences among the two groups (Table 4). Two patients (4.6%) had an all-cause death in the non-cryoICE™ cohort. One (1.7%) patient had a nonfatal MI that was medically treated. No patient had a cardiac death or surgical reintervention. MACCE was 1 (1.7%) in the cryoICE™ cohort and 2 (4.6%) in the non-cryoICE™. In addition, 1 (1.7%) patient experienced non-fatal stroke in the cryoICE™ cohort.

**Table 4.** **Follow-up clinical outcomes**.

Follow-up clinical outcomes	cryoICE™ Patients	Non-cryoICE™ Patients	*p*-value	All patients
	N = 60	N = 43		N = 103
Cardiac readmission, n (%)	5 (8.3%)	8 (18.6%)	0.126	13 (13.0%)
All-cause death, n (%)	0	2 (4.6%)	0.093	2 (1.9%)
Cardiac death, n (%)	0	0	1.000	0
Repeat revascularization, with stents in non-target vessels n (%)	2 (3.3%)	1 (2.3%)	0.757	3 (2.9%)
Myocardial infarction, n (%)	1 (1.7%)	0	0.392	1 (0.9%)
Stroke, n (%)	1 (1.7%)	0	0.392	1 (0.9%)
MACCE, n (%)	1 (1.7%)	2 (4.6%)	0.093	2 (1.8%)

MACCE, major adverse cardiovascular and cerebrovascular events.

### 3.5 Pain Questionnaire for the cryoICE™ Cohort

Pain questionnaire elicited important findings, including a mean value of incisional pain at hospital discharge of 1.5 (scale 0–10) and at follow-up of 0.69 (scale 0–10) (Table 5). The mean value of elicited pain with movement at hospital discharge was 1.15 (scale 0–10). Mean values of skin numbness at hospital discharge were 1 (scale 0–10) and 0.57 (scale 0–10) at follow-up. Mean values of skin hypersensitivity at hospital discharge were 1.1 (scale 0–10) and 0.9 (scale 0–10) at follow-up. Mean values of pain affecting the quality of sleep and pain at breathing were both 0.34 (scale 0–10) upon hospital discharge and follow-up. Most of the patients that experienced pain at hospital discharge and follow-up described the quality of pain as stabbing pain.

**Table 5.** **Pain questionnaires outcomes**.

Pain questionnaires outcomes	Patients N = 60
	Mean values
Pain at discharge (scale 0–10)	1.5
Pain at follow-up (scale 0–10)	0.69
Skin numbness at discharge (scale 0–10)	1
Skin numbness at follow-up (scale 0–10)	0.57
Skin hypersensitivity at discharge (scale 0–10)	1.1
Skin hypersensitivity at follow-up (scale 0–10)	0.9
Pain affecting sleep at discharge (scale 0–10)	0.34
Pain affecting sleep at follow-up (scale 0–10)	0.34
Pain affecting breathing at discharge (scale 0–10)	0.34
Pain affecting breathing at follow-up (scale 0–10)	0.34
Pain with movement at discharge (scale 0–10)	1.15

### 3.6 In-Hospital Drug Use Outcomes

With respect to intra-operative drug use, including fentanyl, ketorolac and lidocaine, there were no significant differences among the two cohorts (Table 6). Postoperatively, hydromorphone IV use was significantly lower in the cryoICE™ vs non-cryoICE™ cohorts, 6.0 ± 7.0 vs 10.8 ± 14.1 mg (*p* = 0.025) (Table 6). Intraoperatively, there was an overall 27% increase of total opioid consumption while postoperatively there was a total reduction of 36% of opioid consumption in the cryoICE™ compared to non-cryoICE™ cohort. During the entire hospital LOS there was a 49% opioid consumption in the cryoICE™ compared to non-cryoICE™ cohort (Table 7).

**Table 6.** **In-hospital drugs use**.

In-hospital drugs use	cryoICE™ Patients	Non-cryoICE™ Patients	*p*-value	All patients
	N = 60	N = 43		N = 103
Intra-operative drugs				
Fentanyl IV mcg mean ± SD	205.8 ± 82.9	206.4 ± 106.2	0.977	206.1 ± 92.8
Ketorolac IV mg mean ± SD	12.0 ± 11.6	16.4 ± 13.8	0.085	13.8 ± 12.7
Lidocaine IV mg mean ± SD	21.3 ± 62.6	9.5 ± 15.9	0.229	16.4 ± 49.1
Post-operative drugs				
Oxycodone oral mg mean ± SD	27.8 ± 47.4	23.3 ± 27.8	0.584	23.3 ± 40.3
Hydromorphone IV mg mean ± SD	6.0 ± 7.0	10.8 ± 14.1	0.025	8.0 ± 10.8

IV, intravenous.

**Table 7.** **Overall in-hospital drugs use**.

In-hospital drugs use	cryoICE™	Non-cryoICE™	Reduction in opioid consumption in the cryoICE™ cohort %
	N = 60	N = 43	
Intra-operative IV morphine equivalent dose mg (fentanyl + ketorolac)	46.7 ± 63.2	36.7 ± 18.6	27% increase
Post-operative IV morphine equivalent dose mg (hydromorphone + oxycodone)	53.8 ± 54.9	83.8 ± 95.9	36%
Total IV morphine equivalent dose mg (fentanyl + hydromorphone + oxycodone)	73.8 + 79.37	144.1 + 118.99	49%

IV, intravenous.

## 4. Discussion

Summary of findings:

(1) The cryoICE™ group evidenced a post-operative reduction in ketorolac IV use.

(2) The cryoICE™ cohort had a lower percentage of opioid use post-operatively and during overall hospital LOS compared to the non- cryoICE™ group.

(3) Post-operative and long-term pain outcomes after cryoICE™ through pain questionnaire evidenced good clinical outcomes with a low grade of incisional pain and numbness.

This analysis provided several novel insights in the fragile CABG population receiving cryoICE™. Firstly, the use of cryoICE™ provides good outcomes in term of intensity of pain, skin numbness and hypersensitivity. Secondly, the total amount of in-hospital opioids markedly decreases (by 49%) with cryoICE™ utilization.

Patients refer different levels of post-operative pain while pain itself is self-determined variable, therefore difficult to precisely quantify. Revealing this heterogeneity of pain provides an impetus to understand the determinants of different outcomes and targets of intervention to ensure that more people have less pain. In this context, extreme skin hypersensitivity or numbness persistence were not observed in our population. This may be related to the correct utilization of the procedure. In addition, the pain score in our study was found to be lower than reported in a previous study comparing allodynia rates in patients who received cryoanalgesia or epidural analgesia [5, 8]. Ju *et al*. [8] reported significantly higher post-operative allodynia-like pain at 6- and 12-months with cryoanalgesia. Another recent study using the same cryoprobe as FROST for Cryo Nerve Block did not find any allodynia complications in treated patients after a median of 529 days (interquartile range 268–637 days); however, the method of assessment was not specified in that study [5, 9].

The heterogeneity of the identified pain trajectories is important because pain and other post-operative recovery domains have mostly been reported as cohort-level average, even in studies evaluating pain at multiple postoperative time points. Opioid-sparing pain management alternatives have been emphasized by both the Enhanced Recovery After Surgery Society guidelines (Class 1 Recommendation to pursue multimodal, opioid-sparing, pain management plan post cardiac surgery [5, 10] and a study using the STS Adult Cardiac Surgery Database, which identified that 12.5% and up to 15.7% of patients become newly persistent opioid users after cardiac and lung surgery, respectively [11].

Altogether, the good results, low event rates and the presence of significant weighted opioids use differences between groups in this analysis suggest the need to weigh not only clinical events but also patients’ preferences (based on the clinician/clinical trials input) in terms of cryoICE™ utilization during the surgical procedure.

## 5. Limitations

This retrospective study was subject to all limitations inherent to a non-randomized study, including potential selection bias regarding which patients underwent left thoracotomy CABG. However, the rigorous propensity-matched analysis limited these biases. In addition, another limitation is the single center/single surgeon data therefore, our analysis necessitates further validation from multicenter studies.

## 6. Conclusions

Excellent clinical outcomes were observed in patients undergoing isolated left thoracotomy CABG with CryoICE™ utilization, including a very low incidence of post-operative pain, numbness, and hypersensitivity for all comers. In addition, the use of the device led to a substantial reduction of cumulative opioid use.

## Data Availability

The data that support the findings of this study are available upon reasonable request to Dr. Serge Sicouri, pending institutional approval.

## Supplementary Material

Supplementary material associated with this article can be found, in the online version, at https://doi.org/10.31083/j.rcm2406182.
