# Supplementary material for: Clinical Outcomes of Cryo Nerve Ablation Technique for Pain Management: An Exploratory Study in Patients Undergoing Left Thoracotomy Coronary Artery Bypass Grafting
Source: Rev Cardiovasc Med. 2023 Jun 25;24(6):182. doi: 10.31083/j.rcm2406182 (PMC11264104; doi:10.31083/j.rcm2406182)
Supplement: Supplementary file 1 [file 2153-8174-24-6-182-s1.zip › 2153-8174-24-6-182-s1/Supplemental Document (3)-How the device works, indications and complications.docx]

How does the device work?

Our nerve has two main parts, an outer protective structure, and the actual nerve that senses pain. By freezing the nerve at a specific temperature, it is disabled without damaging the protective structure, therefore allowing it to regrow right back.

Indications for Cryo Nerve Block

- Thoracotomy
- Lung surgery
  - Pneumonectomy, Lobectomy, Wedge Resection, Segmentectomy
- Lung Transplant
- Pectus Excavatum Repair (NUSS Procedure)
- Rib Fracture Repair
- Esophagectomy
- Minimally invasive cardiac surgery

Complications

- Pneumothorax
- Hypoalgesia
- Hyperalgesia
- Allodynia
- Prolonged numbness greater than 3 months
