# Supplementary material for: Clinical Outcomes of Cryo Nerve Ablation Technique for Pain Management: An Exploratory Study in Patients Undergoing Left Thoracotomy Coronary Artery Bypass Grafting
Source: Rev Cardiovasc Med. 2023 Jun 25;24(6):182. doi: 10.31083/j.rcm2406182 (PMC11264104; doi:10.31083/j.rcm2406182)
Supplement: Supplementary file 1 [file 2153-8174-24-6-182-s1.zip › 2153-8174-24-6-182-s1/Supplemental Document (4)-Questionnaire.docx]

Questionnaire

1. By the time you left the hospital following cardiac surgery procedure, how would you rate your pain at the surgical incision site, from a scale from 0-10, where 0 is no pain and 10 is extreme pain?
2. How would you rate your pain at this moment at the surgical incision site from a scale from 0-10, where 0 is no pain and 10 is extreme pain?
3. Did you experience any pain with movement by the time you left the hospital after cardiac surgery procedure? How would you rate your pain at that time at the surgical incision site, from a scale from 0-10, where 0 is no pain and 10 is extreme pain?
4. By the time you left the hospital following cardiac surgery procedure, how would you rate your skin numbness at the surgical incision site from a scale from 0-10, where 0 is no numbness and 10 is severe numbness?
5. How would you rate your numbness at the surgical incision site now, from a scale from 0-10, where 0 is no numbness and 10 is severe numbness?
6. By the time you left the hospital following cardiac surgery procedure, how would you rate your skin sensitivity at the surgical incision site, from a scale from 0-10, where 0 is no sensitivity and 10 is extremely sensitive?
7. How would you rate your skin sensitivity at the surgical incision site, from a scale from 0-10, where 0 is no sensitivity and 10 is extremely sensitive?
8. By the time you left the hospital following cardiac surgery procedure, was the pain interfering with your quality of sleep? In case it was interfering, how bad was it, from a scale from 1-10, where 1 is mild interference and 10 is no sleep at all?
9. Is the pain now interfering with your quality of sleep? In case it is interfering, how bad is it from a scale from 1-10, where 1 is mild interference and 10 is severe interference (no sleep at all)?
10. By the time you left the hospital following cardiac surgery procedure, was the pain interfering with your breathing? In case it was interfering, how bad was it from a scale from 1-10, where 1 is mild interference and 10 is severe interference?
11. Is the pain now interfering with your breathing? In case it is interfering, how bad is it from a scale from 1-10, where 1 is mild interference and 10 is severe interference?
