# Supplementary material for: Clinical Outcomes of Cryo Nerve Ablation Technique for Pain Management: An Exploratory Study in Patients Undergoing Left Thoracotomy Coronary Artery Bypass Grafting
Source: Rev Cardiovasc Med. 2023 Jun 25;24(6):182. doi: 10.31083/j.rcm2406182 (PMC11264104; doi:10.31083/j.rcm2406182)
Supplement: Supplementary file 1 [file 2153-8174-24-6-182-s1.zip › 2153-8174-24-6-182-s1/Supplemental document 2-Covariates and exposures, and surgical technique.docx]

**1.1 Covariate and Exposures**

Covariate included age, gender, race, STS risk of mortality, creatinine level, comorbidities such as preoperative dialysis, smoking, chronic obstructive pulmonary disease, hypertension, dyslipidemia, cerebrovascular disease, peripheral vascular disease, liver disease and diabetes. Preoperative cardiothoracic history included mediastinal radiation, prior percutaneous coronary intervention, prior coronary artery bypass grafting (CABG), prior myocardial infarction, prior valve surgery, atrial fibrillation, ejection fraction, number of diseased vessels, left main coronary artery stenosis, severe proximal left anterior descending lesion, internal mammary artery, and radial artery graft use. It also included procedure characteristics, including saphenous vein grafts, number of grafts, type of CABG (multi-arterial, total arterial revascularization, and surgery priority). We then collected intra-operative and post-operative outcomes, time in operating room, blood transfusions, extubation in operating room, intensive care unit stay and total length of stay, post-operative stroke, infection, reoperation for bleeding, prolonged ventilation (> 24 hrs), renal failure, dialysis, postoperative atrial fibrillation, and 30-day readmissions.

**1.2 Surgical Procedure**

Robotic-assisted CABG includes the robotic harvest of the internal mammary artery (IMA) and its direct anastomosis to the coronary artery with a small anterior thoracotomy (4 cm) at the site of the camera port. The robotic procedures started with the introduction of 3 ports in the midclavicular/anterior axillary line, in 2nd, 4th and 6th intercostal space. A camera and two lateral arms with surgical instruments are introduced and the surgeon sits on the robotic console while a table side assistant positions the robotic surgical instruments (Intuitive da Vinci® Robotic). The 3D view offered by the robotic platform enhances the visualization of the IMA, lowering the risk of vessel injury and enables the surgeon to harvest a longer IMA graft by means of skeletonization. If a second IMA is required, the surgeon can open the right pleura crossing the mediastinum and accessing the RIMA from the left side chest. Our technique is based on “incision-precision” (12) where the camera port is located at the precise site of the LAD landing zone. By extending the camera port size to a 4 cm skin incision, the surgeon can perform an off-pump coronary anastomosis using a composite off-pump retractor (Medtronic Octopus® Nuvo Tissue Stabilizer) and off-pump technique to complete the anastomosis. In case two arterial grafts are utilized one side of the second arterial graft is anastomosed to the left internal thoracic artery (LITA) (in a Y or T shape anastomosis fashion) and the other side to the coronary targets (diagonal branch, intermediate branch, and obtuse marginal branch) either sequential or end-to-end. In case a vein graft is used, the proximal side is anastomosed to the LITA and the distal side to the other coronary targets.

**1.3 Intra-venous Morphine Conversion Table**

| **Drug** | **Equivalency to 1 mg IV Morphine** | **Conversion Factor** |
| --- | --- | --- |
| Hydromorphone IV | 0.15 mg | 6.7 |
| Fentanyl IV | 10 mCg | 0.1 |
| Oxycodone PO | 2 mg | 0.5 |
| Hydrocodone PO | 3 mg | 0.3 |
| Ketorolac IV | 2.5 mg | 0.4 |
