# Supplementary material for: Clinical Outcomes of Cryo Nerve Ablation Technique for Pain Management: An Exploratory Study in Patients Undergoing Left Thoracotomy Coronary Artery Bypass Grafting
Source: Rev Cardiovasc Med. 2023 Jun 25;24(6):182. doi: 10.31083/j.rcm2406182 (PMC11264104; doi:10.31083/j.rcm2406182)
Supplement: Supplementary file 1 [file 2153-8174-24-6-182-s1.zip › 2153-8174-24-6-182-s1/Supplementary document (1)-Definitions according to the STS.docx]

***Myocardial Infarction***

Myocardial Infarction (MI): (0-24 hours post-op) indicates the presence of a peri-operative MI (0-24 hours post-op) as documented by the following criteria: • The CK-MB (or CK if MB not available) must be greater than or equal to 5 times the upper limit of normal, with or without new Q waves present in two or more contiguous ECG leads. No symptoms required. • (> 24 hours post-op) Indicate the presence of a perioperative MI (> 24 hours post-op) as documented by at least one of the following criteria: 1. Evolutionary ST- segment elevations 2. Development of new Q- waves in two or more contiguous ECG leads 3. New or presumably new LBBB pattern on the ECG 4. The CK-MB (or CK if MB not available) must be greater than or equal to 3 times the upper limit of normal.

***Reoperations (cardiac):***

Operative re-intervention was required for bleeding/ tamponade, valvular dysfunction, graft occlusion and or other complications.

***Sternum infection:***

Indicate whether the patient, within 30 days postoperatively, had a deep sternal infection involving muscle, bone, and/or mediastinum REQUIRING OPERATIVE INTERVENTION. Must have ALL of the following conditions:

1. Wound opened with excision of tissue (I&D) or re-exploration of mediastinum

2. Positive culture

3. Treatment with antibiotics

***Renal failure:***

Indicate whether the patient had acute or worsening renal failure resulting in one or more of the following:

1. Increase of serum creatinine to > 2.0, and 2x most recent preoperative creatinine level.

2. A new requirement for dialysis postoperatively.

***Stroke:***

Indicate whether the patient has a postoperative stroke (i.e., any confirmed neurological deficit of abrupt onset caused by a disturbance in cerebral blood supply) that did not resolve within 24 hours.

***Diabetes:***

A history of diabetes, regardless of duration of disease or need for anti-diabetic agents.

***Complications:***

Indicate whether a post-operative event occurred during the hospitalization for surgery. This includes the entire post-operative period up to discharge, even if over 30 days.
